# Correction: Structural basis of phosphatidylcholine recognition by the C2-Domain of cytosolic phospholipase A_2_α

**DOI:** 10.7554/eLife.75278

**Published:** 2021-11-29

**Authors:** Yoshinori Hirano, Yong-Guang Gao, Daniel J Stephenson, Ngoc T Vu, Lucy Malinina, Dhirendra K Simanshu, Charles E Chalfant, Dinshaw J Patel, Rhoderick E Brown

**Keywords:** Chicken, Human

2019. Hirano Y, Gao Y-G, Stephenson DJ, Vu NT, Malinina L, Simanshu DK, Chalfant CE, Patel DJ, Brown, RE. 2019. Structural basis of phosphatidylcholine recognition by the C2-domain of cytosolicphospholipase A_2_α. *eLife* **8**:e44760. doi: 10.7554/eLife.44760

Published 03 May 2019

Errors have recently come to light regarding the descriptions of the model membranes used in fluorescence resonance energy transfer (FRET) experiments to compare the membrane partitioning of wild-type C2-domain versus Y96F, N65D, and Y96A C2-domain mutants. The errors involve Figures 3B, 3C, and 3D but do not alter the conclusions drawn from the FRET experiments.

At the time of original publication, the model membranes used as energy acceptors in the FRET experiments were described as dansyl-PE/POPC/DHPC (5:45:50) bicelles. Based on more recent experiments performed for a 2020 *Analytical Chemistry* publication (Gao et al., 2020) and further elaborated in a recent (2021) *Bio-protocol* article (Gao et al., 2021), we now know that describing the energy acceptor model membranes as dansyl-PE/POPC/DHPC bicelles is incorrect. In the *Analytical Chemistry* paper, we showed that dilution of POPC-DHPC (50:50) ‘bicelle’ lipid mixes to the low micromolar range, as similarly employed and described in the Methods for the FRET experiments involving the C2-domain and mutants, results in nearly immediate transformation into small POPC unilamellar bilayer vesicles containing little or no DHPC. The transition reflects DHPC’s high aqueous solubility. Thus, corrections have been introduced into the text (Methods) and the labels for the Figures 3B and 3C axes. We also have adjusted the Figure 3C X-axis label from "mM" to "µM" as correctly conveyed in the original Methods section.

The article text and associated panels in Figure 3 have been corrected accordingly.

The text corrections in the Figure 3 legend (changes are underlined) apply to (**B**) and (**C**):

Figure 3. Membrane partitioning of cPLA_2_α C2-domains and cPLA_2_α catalytic activity with point mutated C2-domains in PC binding region. (A) SPR binding isotherms showing point mutant and control protein equilibrium adsorption to immobilized POPC vesicles saturating a L1 sensor chip at 5 μl/min solution flow rates (see Methods). (B) FRET binding isotherms showing the Ca^2+^ dependence of point mutant and control protein (0.5 μM) equilibrium adsorption to POPC/DHPC bicelle-dilution vesicles (4 μM) (see Methods). (C) FRET binding isotherms showing the POPC/DHPC bicelle-dilution vesicle dependence of point mutant and control protein (0.5 μM) equilibrium adsorption at 50 μM Ca^2+^ (see Methods). (D) Relative binding affinity of C2-domain point mutants and control protein obtained for binding isotherms shown in (C). (E) Effect of Y96F, Y96A, N64A, and N65D mutations on dissociation constant (K_s_^A^) of human cPLA_2_α activity. Proteins were purified as described in (Stahelin et al., 2007). Activity was measured as a function of PC molar concentration for 60 min at 37 °C. The PC mole fraction was held constant at 0.285. cPLA_2_α activities (nmol of arachidonic acid released/min/mg of recombinant cPLA_2_α) were collected on eight separate occasions and are presented as n = 4 for Y96F, n = 4 for Y96A, n = 4 for N64A, n = 4 for N65D, and n = 8 for WT. Error = standard deviation. R^2^ values are 0.9021, 09609, 0.9586, 0.9780, and 0.9485 for WT, Y96F, Y96A, N64A, and N65D, respectively. (F) Effect of Y96F, Y96A, N64A, and N65D mutations on allosteric sigmoidal constant (K_half_) of human cPLA_2_α activity. Activity was measured as function of increasing PC mole fractions for 60 min at 37 °C. The PC mole fraction ([PC]/[PC]+[TX-100]) was 0.024 at 50 μM PC, 0.047 at 100 μM PC, 0.069 at 150 μM, 0.091 at 200 μM, 0.13 at 300 μM PC, 0.166 at 400 μM, 0.2 at 500 μM PC, 0.28 at 800 μM PC, 0.37 at 1200 μM PC, and 0.44 at 1600 μM PC. cPLA_2_α activities (nmol of arachidonic acid released/min/mg of recombinant cPLA_2_α) were collected on ten separate occasions and are presented as n = 4 for Y96F, n = 4 for Y96A, n = 4 for N64A, n = 4 for N65D, and n = 4 for WT. Error = standard deviation. R^2^ values are 0.9413, 0.9577, 0.9407, 0.9376, and 0.9761 for WT, Y96F, Y96A, N64A, and N65D, respectively.

The corrected Figure 3 is shown here:

**Figure 1:**
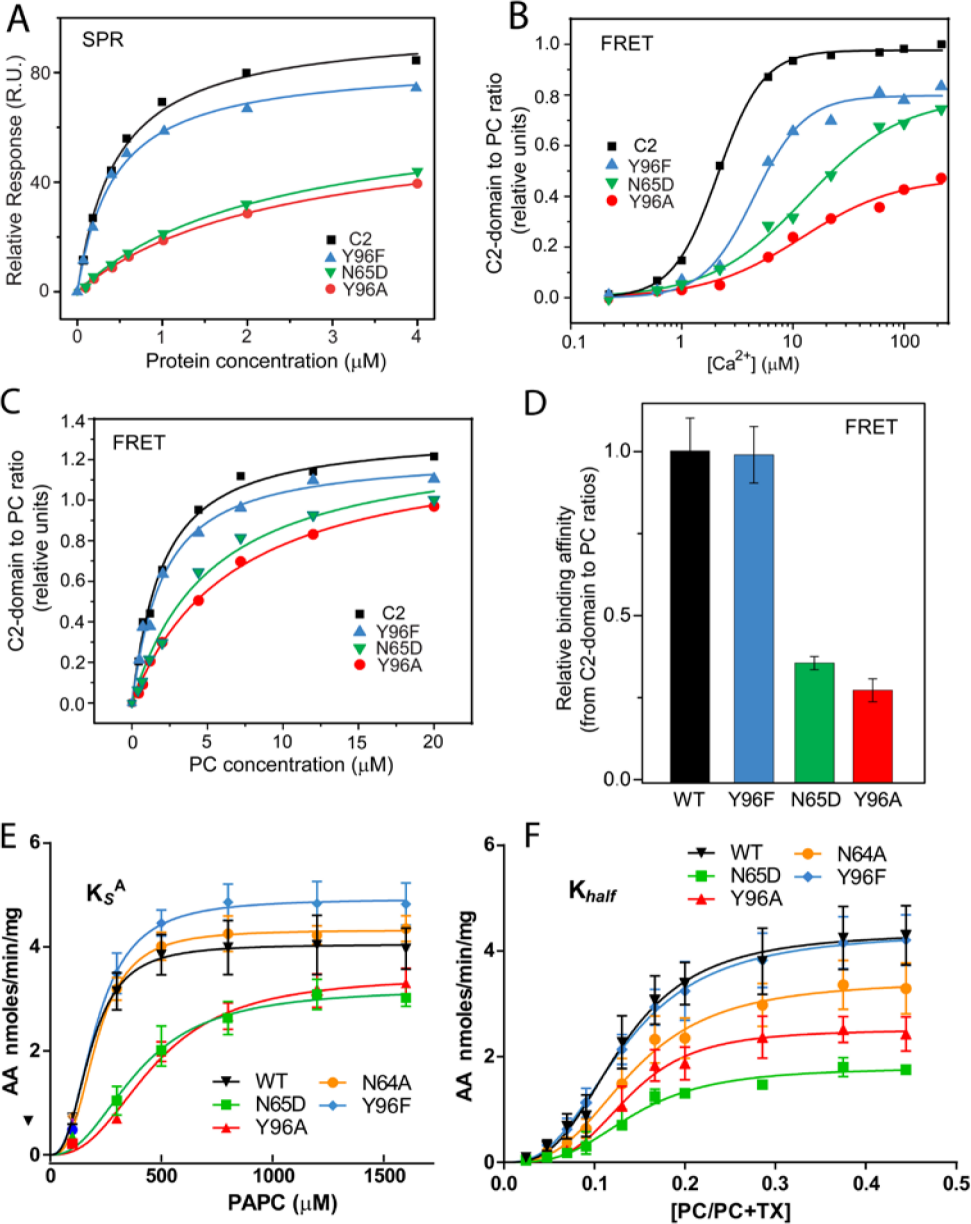


Figure 3, as originally published, is shown here for reference:

**Figure 2:**
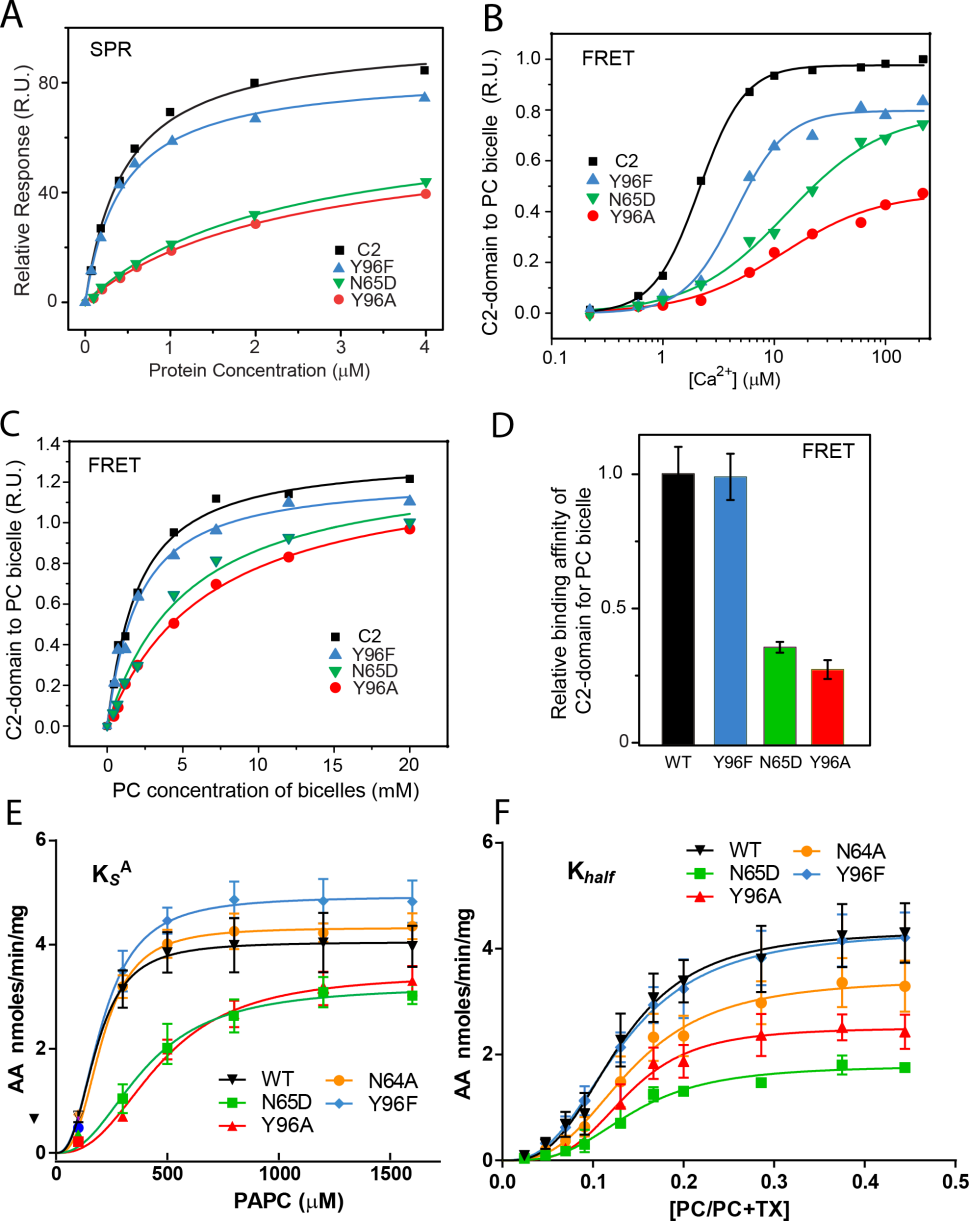


The METHODS subsection, “Point mutant analyses of C2-domain translocation to PC model membranes”, has been corrected by adjusting the text as follows (changes are underlined):FRET measurements were performed using Trp/Tyr emission of C2–domain as energy donor and dansyl-PE-POPC-DHPC (5:45:50) bicelle-dilution vesicles as energy acceptors. Bicelle-dilution vesicles were formed by mixing the POPC, dansyl-PE and DHPC in chloroform, drying under a stream of nitrogen and placing under vacuum for ~2 h, before resuspending in buffer (20 mM Tris, pH 7.5, 150 mM NaCl and 50 µM CaCl_2_). Unilamellar POPC vesicle preparation by POPC/DHPC bicelle mix dilution is detailed in Gao et al., 2020. Binding reactions included C2–domain (0.5 µM) and various amounts of bicelle-dilution vesicles (PC conc. 0.44–20 µM) in 2.5 ml of buffer. In binding reactions assessing calcium dependence (2.5 ml total vol.), the protein and bicelle dilution-vesicle concentrations (0.5 µM and 4 µM, respectively) were held constant while the Ca^2+^ was varied.

The new references (Gao et al., 2020 and Gao et al., 2021) that document the formation of small homogeneous unilamellar POPC bilayer vesicles by dilution of POPC/DHPC bicelle mixtures (0.5 q-value) have now been added to the REFERENCES section. They are:

Gao Y-G, Le LTM, Zhai X, Boldyrev IA, Mishra SK, Tischer A., Murayama T, Nishida A, Molotkovsky JG, Alam A, Brown RE. 2020. Measuring lipid transfer protein activity using bicelle-dilution model membranes. *Anal. Chem*. **92**:3417–3,425 (doi: 10.1021/acs.analchem.9b05523).

Gao Y-G, Le LTM, Alam A, Brown RE. 2021. A simple, straightforward approach for generation of small, stable, homogeneous, unilamellar 1-palmitoyl 2-oleoyl phosphatidylcholine (POPC) bilayer vesicles. *Bio-protocol* 11:e2104322 (in press; pub. date:12/20/2021).
