# Supplementary material for: Structural basis of phosphatidylcholine recognition by the C2–domain of cytosolic phospholipase A2α
Source: eLife. 2019 May 3;8:e44760. doi: 10.7554/eLife.44760 (PMC6550875; doi:10.7554/eLife.44760)
Supplement: Figure 3—source data 1. [file elife-44760-fig3-data1.pdf]

Figure 3 - source data 1

Membrane partitioning data for cPLA2α C2-domains mutated in PC binding region

Figure 3A (SPR data)

C2 domain conc. (μM)	C2 WT	Y96F	Y96A	N65D
0.00	0.00	0.00	0.00	0.00
0.10	10.72	10.43	1.89	2.63
0.20	26.35	23.37	4.12	5.81
0.40	44.29	43.70	9.18	9.90
0.60	57.12	51.53	13.41	14.22
1.00	68.78	58.39	18.31	20.94
2.00	79.89	67.15	28.58	32.01
4.00	84.69	75.06	39.52	43.39

Figure 3B (FRET data)

[Ca2+ conc.] (μM)	C2 WT	Y96F	Y96A	N65D
0.00	0.00	0.00	0.00	0.00
0.22	0.01	0.02	-0.01	-0.01
0.60	0.07	0.03	0.03	0.03
1.00	0.15	0.07	0.03	0.05
2.20	0.52	0.13	0.05	0.11
6.00	0.87	0.54	0.16	0.28
10.00	0.93	0.66	0.24	0.32
22.00	0.96	0.70	0.31	0.48
60.00	0.97	0.81	0.36	0.67
100.00	0.98	0.78	0.43	0.69
220.00	1.00	0.84	0.47	0.74

Figure 3C (FRET data)

[Lipid conc.] (μM)	C2 WT	Y96F	Y96A	N65D
0.00	0.00	0.00	0.00	0.00
0.44	0.21	0.21	0.05	0.06
0.72	0.40	0.38	0.09	0.10
1.20	0.44	0.38	0.21	0.21
2.00	0.66	0.64	0.30	0.29
4.40	0.95	0.84	0.51	0.64
7.20	1.12	0.96	0.70	0.81
12.00	1.14	1.10	0.83	0.92
20.00	1.22	1.11	0.97	1.00

Figure 3 - source data 1

Figure 3D (FRET data)

	Affinity	SD
WT	1.00	0.10
Y96A	0.27	0.04
Y96F	0.99	0.09
N65D	0.35	0.02
