# Supplementary material for: Structural basis of phosphatidylcholine recognition by the C2–domain of cytosolic phospholipase A2α
Source: eLife. 2019 May 3;8:e44760. doi: 10.7554/eLife.44760 (PMC6550875; doi:10.7554/eLife.44760)
Supplement: Figure 4—source data 1. [file elife-44760-fig4-data1.pdf]

**Figure 4 -source data 1**

cPLA2α C2-domain binding affinity for phosphoglyceride and sphingomyelin (SM) vesicles

Figure 4B

| Phosphoglyceride | Relative Response (R.U.) | SD |
|---|---|---|
| PC | 68.78 | 7.14 |
| PS/PE | 11.6 | 2.18 |
| PS | 4.74 | 1.89 |
| PA | 7.05 | 2.18 |
| PG | 9.77 | 2.24 |
| PI | 8.99 | 2.07 |
| PC/PS | 61.54 | 5.54 |
| PC/PE | 63.48 | 4.37 |

Figure 4C

| C2 domain conc. (μM) | POPC Relative Response (R.U.) | 18:1 SM Relative Response (R.U.) |
|---|---|---|
| 0 | 0 | 0 |
| 0.1 | 10.72 | 1.5 |
| 0.2 | 26.35 | 3.61 |
| 0.4 | 44.29 | 7.85 |
| 0.6 | 59.12 | 12.42 |
| 1 | 69.78 | 19.01 |
| 2 | 79.89 | 42.34 |
| 4 | 82.69 | 64.95 |

Figure 4D

| C2 domain | 18:1 SM Relative Response (R.U.) | SD |
|---|---|---|
| WT | 23.92 | 5.38 |
| Y96F | 16.04 | 3.72 |
| Y96A | 7.64 | 1.95 |
| N65D | 9.41 | 2.54 |
